# Dynamic Links Between Daily Positive Parenting and Adolescent Well‐Being: The Moderating Role of Daily Adolescent Emotion Regulation

**DOI:** 10.1002/jad.70179

**Published:** 2026-05-14

**Authors:** Lan Chen, Gregory M. Fosco

**Affiliations:** ^1^ Human Development and Family Studies The Pennsylvania State University University Park Pennsylvania USA; ^2^ Edna Bennett Pierce Prevention Research Center Pennsylvania State University University Park Pennsylvania USA

**Keywords:** adolescent well‐being, emotion regulation, intensive longitudinal methods, parents' use of positive behavior support

## Abstract

**Introduction:**

Well‐being tends to decline during adolescence, and recent evidence documents that it also fluctuates within adolescents from day to day. Parents' use of positive behavior support is a well‐established positive parenting strategy for promoting children's development, yet there is limited research investigating its implications during the adolescent developmental period. The current study evaluated the daily linkage between parents' use of positive behavior support and adolescents' well‐being, and whether daily adolescent emotion regulation moderated these associations.

**Methods:**

This study included a sample of 135 parents (91.9% female; *M*_*age*_ = 47.65, SD_*age*_ = 6.17) and their adolescents (54.8% female; *M*_*age*_ = 15.56, SD_*age*_ = 1.16), who completed a 21‐day daily diary protocol. Participants were recruited across the United States between 2022 and 2024.

**Results:**

Multilevel model results indicated that daily variations in adolescent emotion regulation moderated the relation between parents' use of positive behavior support and adolescent well‐being. Specifically, daily parents' use of positive behavior support was linked to better well‐being only on days when adolescents reported lower emotion regulation.

**Conclusions:**

Our study provides novel support for a support needs perspective—that on days when adolescents are struggling more with managing their emotions, parents' use of positive behavior support is most beneficial for adolescent well‐being. These findings highlight the importance of examining parenting effect heterogeneity at the daily timescale.

Adolescence is a developmental period characterized by an increased risk for a range of mental health problems (Blakemore 2019). At the same time, subjective well‐being, including life satisfaction and positive affect, declines during this period (Goldbeck et al. 2007; Griffith et al. 2021). Mental health problems and low subjective well‐being during adolescence have long‐term implications for psychopathology risk, low academic achievement, and poor physical health in the adult years (Hoyt et al. 2012; Suldo et al. 2011; Suldo and Huebner 2004). Well‐being is conceptualized as both the absence of mental health problems and the presence of positive subjective well‐being; as these represent complementary rather than opposing dimensions (Greenspoon and Saklofske 2001; Karademas 2007). Individuals low in mental health problems may still be at risk for negative outcomes without subjective well‐being, highlighting the importance of considering both dimensions simultaneously (Antaramian et al. 2010; Magalhães 2024).

Although adolescent well‐being is often treated as a stable, trait‐like characteristic, emerging research demonstrates that it also exhibits state‐like qualities, fluctuating from day to day (Fosco and Lydon‐Staley 2019; Fosco and Lydon‐Staley 2020). Additionally, many studies have aggregated various aspects of negative mood to reflect daily distress. However, depressed, angry, and anxious moods may have distinct mechanisms (Chen et al. 2024). Thus, the present study examined daily factors that promote adolescent well‐being by examining both distress (i.e., depressed, angry, and anxious mood) and subjective well‐being (i.e., positive mood and life satisfaction).

## 1 Parents' Use of Positive Behavior Support

Previous studies have documented that positive parenting is a robust predictor of better adolescent well‐being (Guevara et al. 2021; Smokowski et al. 2015); yet, in early and middle adolescence, families exhibit normative declines in positive parenting (Dishion et al. 2004). This decline underscores the tenuous nature of positive parenting during this period; moreover, it points to a risk for adolescent disengagement from the family, as parental involvement and awareness of adolescents' activities decline, and adolescents feel less connected to their parents (Fosco and LoBraico 2019). In turn, adolescent disengagement from the family is a risk factor for antisocial behavior and depression (Dishion et al. 2004; Fosco et al. 2012; Fosco and LoBraico 2019). Corresponding declines in parenting quality and adolescent well‐being during the adolescent years make this developmental period a critical area for study.

Research on positive parenting during adolescence has focused largely on parental monitoring, limit setting, parent‐adolescent relationship quality, and autonomy support (Dishion et al. 2004). The current study focused on parents' use of *positive behavior support*—less studied in adolescence—defined as a set of positive parenting skills that includes structuring proactive environments to encourage positive behaviors and using praise or compliments to reinforce desirable behaviors (Fosco and LoBraico 2019; Lunkenheimer et al. 2008; Waller et al. 2015). Positive behavior support is conceptually and empirically distinct from other key parenting constructs in that it promotes positive behaviors through reinforcement rather than supervision (monitoring), constraint (limit setting), or volitional support (autonomy support); confirmatory factor analyses have demonstrated that these dimensions form separate factors with unique predictive validity for adolescent outcomes (Dishion et al. 2006).

Positive behavior support is a core component of most evidence‐based parenting interventions (Dishion et al. 2008; Lunkenheimer et al. 2008; Van Ryzin et al. 2016; Waller et al. 2015), with longitudinal evidence demonstrating its benefits for reducing children's problem behaviors and improving academic achievement (Dishion et al. 2008; Gill et al. 2008; Lunkenheimer et al. 2008; Shaw et al. 2006; Waller et al. 2015). Despite documented benefits for children's development, there is limited research on the role of parents' use of positive behavior support during adolescence. Family‐based intervention programs incorporating positive behavior support have shown a significant positive impact on adolescent outcomes, including reduced family conflict, deviant peer affiliations, and substance use; yet these studies tested entire intervention packages rather than isolating the specific effect of positive behavior support (Fosco et al. 2016; Van Ryzin et al. 2012). Emerging daily diary research has demonstrated that on days when parents use more positive behavior support, adolescents report feeling closer to parents and experiencing lower negative affect (Fosco and LoBraico 2019; Wu et al. 2025), but relations with other aspects of well‐being remain unclear. Thus, the current study addresses this gap by examining how parents' use of positive behavior support is linked with adolescent well‐being.

### 1.1 Daily Relations Between Positive Behavior Support and Adolescent Well‐Being

Parenting processes operate across multiple timescales (Boele et al. 2020). Historically, research on positive behavior support parenting has largely focused on between‐person differences (i.e., how parenting varies across parents) and their rank‐order associations with adolescents' outcomes; or has used conventional longitudinal designs to capture macro‐level patterns across months or years (e.g., Waller et al. 2015). However, recent work using daily diary designs has documented that parents' use of positive behavior support exhibits meaningful within‐person daily fluctuations (Chen et al. 2025; Fosco and LoBraico 2019; Li and Zheng 2025). Past work has established meaningful within‐person associations between parenting and adolescent well‐being (Boele et al. 2020; Bülow et al. 2022; Fosco et al. 2021; Neubauer et al. 2021; Van Der Kaap‐Deeder et al. 2023; Wu et al. 2025). Critically, lagged analyses from daily diary and ecological momentary studies—including those explicitly accounting for bidirectional effects—indicate that parenting and parent‐adolescent interactions prospectively predict adolescent well‐being (Bülow et al. 2022; Fosco et al. 2025; LoBraico et al. 2020).

Parenting processes at a between‐person level may not generalize to the within‐person level (Boele et al. 2020; Hunter et al. 2024; Molenaar 2004). Building on evidence that parents' use of positive behavior support and adolescent well‐being both demonstrate considerable day‐to‐day variability (e.g., Fosco et al. 2019; Fosco and LoBraico 2019; Fosco and Lydon‐Staley 2019), there is a need to study daily, within‐person variability in positive behavior support and its relations with daily adolescent well‐being. Indeed, within‐person variability in parents' use of positive behavior support is reliably linked with adolescents' feelings of closeness to their parents (Fosco and LoBraico 2019). Moreover, studying parenting at the daily timescale is meaningful because daily processes accumulate over time to shape long‐term resilience (Bai and Repetti 2015). For example, day‐to‐day variation in adolescents' responses to parents' use of positive behavior support has been linked to later antisocial behavior and substance use (Fosco and LoBraico 2019). Thus, the current study examined the role of daily parents' use of positive behavior support in shaping adolescents' daily well‐being.

### 1.2 Daily Adolescent Emotion Regulation as a Moderator

Individuals differ in their response to the same parenting behaviors (Boele et al. 2020). Existing work has demonstrated that individual differences in emotion regulation shape the overall strength of parenting effects at macro timescales (Belsky et al. 2007); for instance, children and adolescents with poorer emotion regulatory capacity tend to be more sensitive to negative parenting, showing steeper links with maladjustment (Creavey et al. 2018; Skowron et al. 2014). More recently, daily diary research has demonstrated that heterogeneity in parenting effects also exists at the within‐person level (Bülow et al. 2022; Janssen et al. 2021); for example, the daily associations between parenting and adolescent life satisfaction differ meaningfully by adolescents' average levels of emotion regulation (Chiang et al. 2023). However, this work has yet to identify what explains within‐person variability in these daily parenting effects; that is, why the same parenting behavior matters more on some days than on others for the same adolescent.

Recent developments in emotion regulation research have led to recognition that individuals' emotion regulation exhibits dynamic properties, and can vary within individuals across time and situations (Cole and Hollenstein 2018; Gross 2015; Gross et al. 2006; Tamir 2021). In addition to serving as a marker of individual differences, accumulating evidence from daily diary and ecological momentary assessment studies now document that individuals differ from day to day in their emotion regulation (Blanke et al. 2020; Boemo et al. 2022; Koval et al. 2023; Silk et al. 2003; Vanderlind et al. 2022). Integrating early work documenting emotion regulation as an individual difference factor with this dynamic view of emotion regulation calls for a better understanding of how day‐to‐day fluctuations in emotion regulation within persons may serve as a proximal context that modifies the link between parenting and adolescent well‐being. To do so, we examined whether a core facet of daily emotion regulation—adolescents' subjective sense of their ability to control or regulate emotions in daily life—explained heterogeneity in the within‐person relation between daily parenting and adolescent well‐being (Gratz and Roemer 2004; Sheppes et al. 2014). To our knowledge, the current study is among the first to examine whether adolescents' day‐to‐day changes in emotion regulation explain differential benefits from daily parents' use of positive behavior support.

Two competing perspectives support hypotheses for how daily emotion regulation shapes parenting effects. A *resource amplification perspective* draws on evidence that emotion regulation requires cognitive and attentional resources (Gross 2015; Sheppes et al. 2014) and suggests that on days when adolescents feel more in control of their emotions, they may have greater available resources to recognize and utilize positive behavior support. That is, higher daily emotion regulation may amplify the benefits of parental support. Alternatively, a *support needs perspective* suggests that fluctuations in emotion regulation reflect changing emotional needs and receptiveness to parental input, such that adolescents may interpret the same parenting behavior differently depending on their regulatory state on a given day (Soenens et al. 2015). On days when adolescents experience poorer emotion regulation, they may be more emotionally vulnerable and therefore more receptive to direct parental support and encouragement, making positive behavior support particularly beneficial for well‐being. In addition, on days when adolescents report lower emotion regulation, they may also experience greater emotional distress, leaving greater room for improvement in response to positive parenting compared with days when well‐being is already high. Thus, the support needs perspective suggests the opposite prediction from the resource amplification perspective: that when youth have the lowest regulatory functioning, they may benefit most from external support.

### 1.3 The Current Study

Adolescent well‐being fluctuates meaningfully from day to day (Fosco and Lydon‐Staley 2020), highlighting the importance of identifying daily‐level factors associated with well‐being. Positive parenting is robustly associated with adolescent well‐being, yet little is known about one salient form—parents' use of positive behavior support—during adolescence. The first aim of this study was to evaluate whether daily fluctuations in parents' use of positive behavior support were associated with daily changes in adolescent well‐being using daily diary methods. We expected that on days when parents' positive behavior support was higher than usual, adolescents would report better well‐being, as indicated by lower levels of depressed, angry, and anxious mood, and higher levels of positive mood and life satisfaction. Building on recent evidence that emotion regulation itself fluctuates meaningfully from day to day (e.g., Blanke et al. 2020), the second aim was to evaluate whether daily fluctuations in adolescent emotion regulation further moderated the daily associations between positive behavior support and adolescent well‐being. Given the novelty of this within‐person moderation question, the moderating roles of both average and daily emotion regulation were treated as exploratory.

## 2 Methods

### 2.1 Participants

The current study used data from 135 parent‐adolescent dyads participating in the Everyday Relationships in Adolescence (ERA) study. Eligibility criteria were that the adolescent and parent: (1) had lived together for at least 1 year; (2) had access to the internet to complete online surveys; (3) were fluent in English; (4) the adolescent was in 9th, 10th, or 11th grade at the beginning of the study. This study was approved by the Penn State Institutional Review Board (protocol #15632; Everyday Relationships in Adolescence).

Adolescent participants (*M*_*age*_ = 15.56, SD_*age*_ = 1.16, range = 13–18) reported their sex assigned at birth as 54.8% female (*n* = 74) and 45.2% male (*n* = 61). Parent participants reported their sex assigned at birth as 91.9% female (*n* = 124) and 8.1% male (*n *= 11). Annual household incomes ranged from less than $10,000 (2.2%) to $125,000 or more (48.1%), with a median income of $100,000–$124,999 per year. Descriptive statistics for all study variables are presented in Table 1.

**Table 1:** Descriptive characteristics of adolescent and parent participants (*N* = 135 Dyads).

Characteristic	*n*	%
Adolescents		
Age (*M* = 15.56, SD = 1.16, Range = 13–18)		
Sex assigned at birth		
Female	74	54.8
Male	61	45.2
Gender identity		
Woman	67	49.6
Man	60	44.4
Gender nonconforming	2	1.5
Non‐binary	1	0.7
Trans male	1	0.7
Not listed	2	1.5
Declined to answer	2	1.5
Race		
White	117	86.7
Black/African American	18	13.3
Filipino	1	0.7
Asian Indian	3	2.2
Korean	1	0.7
Other Asian	2	1.5
Other race	1	0.7
Multiracial	8	5.9
Ethnicity		
Not Hispanic/Latino/Latinx	125	92.6
Mexican/Mexican American/Chicano	2	1.5
Puerto Rican	3	2.2
Other Hispanic/Latino/Latinx	5	3.7
Parents		
Sex assigned at birth		
Female	124	91.9
Male	11	8.1
Gender identity		
Woman	123	91.1
Man	10	7.4
Gender nonconforming	1	0.7
Declined to answer	1	0.7
Relationship to adolescent		
Mother	124	91.9
Father	10	7.4
Aunt	1	0.7
Race		
White	118	87.4
Black/African American	17	12.6
American Indian/Alaska Native	1	0.7
Asian Indian	2	1.5
Vietnamese	1	0.7
Korean	1	0.7
Japanese	1	0.7
Other race	2	1.5
Multiracial	8	5.9
Ethnicity		
Not Hispanic/Latino/Latinx	134	99.3
Other Hispanic/Latino/Latinx	1	0.7
Marital status		
Married	101	74.8
Living together	4	3.0
Separated	4	3.0
Divorced	12	8.9
Single	14	10.4

*Note:* Some participants selected more than one racial category and are reflected as multiracial. Percentages may not total 100 due to rounding or multiple responses.

### 2.2 Procedures

Participants were recruited in collaboration with schools. After establishing eligibility, participants attended a Zoom onboarding session where parental consent and adolescent assent were obtained electronically, household information was collected, identities were verified, and participants were instructed to complete surveys independently and not share their responses with one another. Both parents and adolescents completed baseline surveys followed by 21 consecutive daily surveys. Separate survey links were sent directly to each participant each morning via text message or email according to their preference, and each survey included a reminder at the start to complete the survey independently. In these daily surveys, participants were asked to report on their experiences from the previous day. Surveys were sent at 6:00 am and closed at 2:00 pm, with reminders sent between 10:00 am and 12:00 pm through text message or email. Adolescents completed an average of 86.1% ($M_{\mathrm{Adolecsent}}$ = 18.34, $SD_{\mathrm{Adolecsent}}$ = 4.16) and parents completed 93.4% ($M_{\mathrm{Parent}}$ = 20.04, $SD_{\mathrm{Parent}}$ = 2.18) of daily surveys. Participants were compensated $25 for the baseline survey. For daily surveys, compensation was based on each week's completion rate using an escalating approach. Specifically, participants earned $2.50 for each of the first four surveys completed per week and $5.00 for each additional survey, up to $25 per week (maximum $75 for daily surveys).

### 2.3 Measures

Reliability was estimated using *R*_*C*_ for within‐person consistency across days and *R*_*1F*_ for between‐person reliability based on daily averages (Cranford et al. 2006). For single‐item assessments, reliability estimates could not be computed. All measures used a sliding scale ranging from 0 (None of the Time) to 10 (All of the Time) in 0.1 increments.

### 2.4 Daily Positive Behavior Support Parenting

Each day, parents were asked to report their positive behavior support using two items: “I let [CHILD NAME] know when he/she was doing a good job with something” and “I praised or complimented [CHILD NAME] for good behavior.” Items were averaged to create a daily positive behavior support score, with higher scores indicating greater use of positive behavior support. The inter‐item correlations were significant at both the within‐ and between‐person levels (*r* = 0.69, *p* < 0.01, and *r* = 0.84, *p* < 0.01, respectively). The scale exhibited good within‐person and between‐person reliabilities (*R*_*C*_ = 0.82, *R*_*1F*_ = 0.87). Between‐person levels of positive behavior support were moderately associated with parental warmth (*r* = 0.53, *p *< 0.01), supporting convergent validity. This measure has been used in prior studies with a different sample (Chen et al. 2025; Fosco et al. 2025; Fosco and LoBraico 2019).

### 2.5 Daily Adolescent Emotion Regulation

Each day, adolescents' subjective sense of emotion regulation was assessed using a single item adapted from the Difficulties in Emotion Regulation Scale (Gratz and Roemer 2004) to fit a daily timescale: “Did you feel in control of your emotions?” This item captures adolescents' subjective perception of control over their emotional responses, consistent with conceptualizations emphasizing individuals' felt sense of emotional control (Gratz and Roemer 2004).

### 2.6 Daily Adolescent Mood

Each day, adolescents reported each daily mood using a single‐item assessment adapted from the Profile of Mood States (Curran et al. 1995). Adolescents rated each mood item using the prompt, “How much of the time yesterday did you feel…” with items corresponding to angry mood (angry or annoyed), depressed mood (sad or depressed), anxious mood (anxious or worried), and positive mood (happy).

### 2.7 Daily Adolescent Life Satisfaction

Each day, adolescents' life satisfaction was measured using a single item: “All things considered, I was satisfied with my life.”

### 2.8 Analytic Plan

First, we conducted a variance decomposition wherein participant ID predicted each daily variable, as indicated by the intraclass correlation coefficient (ICC). The ICC was calculated as between‐person variance divided by total variance $\left(\mathrm{ICC}=\frac{\sigma_{\mathrm{between}}^{2}}{\sigma_{\mathrm{within}}^{2}+\sigma_{\mathrm{between}}^{2}}\right)$. Reverse ICC (i.e., 1‐ICC) values above 20% indicated meaningful within‐person variation (Bolger and Laurenceau 2013). Next, we calculated between‐ and within‐person variables for daily parents' use of positive behavior support and adolescent emotion regulation (Bolger and Laurenceau 2013). For between‐person variables, we computed each person's average score across all study days and then calculated the sample mean by averaging these individual scores. We centered the between‐person variables at the sample mean; a positive score indicates that a person's average score is above the sample mean. Within‐person variables were person‐mean centered by subtracting each person's average score from their score on a given day, such that positive values reflect days when the variable is higher than the person's average.

In the first set of analyses, we conducted multilevel models to examine the association between daily parents' use of positive behavior support and daily adolescent well‐being, using 21 days of observations nested within individuals. We controlled for study days (0 = Day 10.5; Bolger and Laurenceau 2013), school day (0 = weekend, 1 = school‐day), prior‐day well‐being outcome to account for autoregressive effects, between‐person levels of positive behavior support, adolescent age, adolescent sex (female = 0, male = 1), and family income. In the second set of analyses, we incorporated a within‐person interaction term to test the moderating role of daily adolescent emotion regulation and used Johnson‐Neyman analyses to determine the regions of significance (Johnson and Fay 1950).

In addition, we conducted post hoc cross‐level moderation analyses examining whether between‐person differences in emotion regulation moderated the daily associations. As a sensitivity analysis, we applied false discovery rate (FDR) adjustment to account for potential inflation of Type I error due to testing multiple effects. All analyses were conducted in R Studio (version 4.3.2). Model specifications, formulas, and R code are provided in the Supporting Materials. This study was not preregistered. The data are available from the corresponding author upon reasonable request.

## 3 Results

Table 2 presents descriptive statistics and within‐ and between‐person correlations among study variables. Reverse ICCs for the study variables exceeded the 20% threshold for examining within‐person processes (Bolger and Laurenceau 2013). To test the first hypothesis (see Supporting Materials: Equation 1‐2e), we computed multilevel models to evaluate the relations between positive behavior support and adolescent well‐being. As shown in Table 3, results partially supported the hypothesis. At the between‐person level, adolescents whose parents reported higher average positive behavior support showed lower angry mood (*b* = −0.12, *p* < 0.01), lower depressed mood (*b* = −0.10, *p* < 0.05), higher positive mood (*b* = 0.15, *p* < 0.05), and greater life satisfaction (*b* = 0.15, *p* < 0.05). Average positive behavior support was not significantly associated with anxious mood (*b* = −0.08, *p* = 0.184). At the within‐person level, daily positive behavior support was significantly associated only with lower daily angry mood (*b* = −0.04, *p* < 0.05). Daily positive behavior support was not significantly related to the remaining well‐being indicators (*bs* = −0.03 to 0.04, *ps* = 0.058 to 0.543).

**Table 2:** Descriptive statistics and correlations.

	1	2	3	4	5	6	7
1. Positive Behavior Support	—	0.02	−0.05*	0.01	−0.05*	0.04^†^	0.05*
2. Adolescent Emotion Regulation	0.11**	—	−0.22**	−0.21**	−0.25**	0.32**	0.32**
3. Adolescent Angry Mood	−0.21**	−0.37**	—	0.27**	0.43**	−0.31**	−0.25**
4. Adolescent Anxious Mood	−0.15**	−0.47**	0.62**	—	0.39**	−0.17**	−0.19**
5. Adolescent Depressed Mood	−0.17**	−0.50**	0.70**	0.80**	—	−0.29**	−0.27**
6. Adolescent Positive Mood	0.25**	0.62**	−0.42**	−0.37**	−0.56**	—	0.48**
7. Adolescent Life Satisfaction	0.20**	0.65**	−0.40**	−0.39**	−0.59**	0.80**	—
Within‐person variance (inverse ICC)	47.33%	52.74%	60.31%	45.26%	47.66%	53.28%	41.90%
Mean	6.48	7.30	2.32	2.18	1.62	7.15	7.04
SD	3.14	2.58	2.13	2.44	2.25	2.28	2.72

*Note:* Between‐person (average) correlations are shown below the diagonal; within‐person (daily) correlations are shown above the diagonal.

Abbreviations: ICC, intraclass correlation coefficient; SD, standard deviation.

*p < 0.05; **p < 0.01; ^†^ *p* = 0.056.

**Table 3:** Testing the role of daily parents' use of positive behavior support on adolescent well‐being.

Main effect	Angry Mood		Anxious Mood		Depressed Mood		Positive Mood		Life Satisfaction	
	***B (SE) p***	β	***Est (SE) p***	β	***Est (SE) p***	β	***Est (SE) p***	β	***Est (SE) p***	β
Intercept ($\gamma_{00}$)	3.56 (1.28) 0.005**	0.10	1.11 (2.03) 0.587	0.10	2.28 (1.66) 0.169	0.09	8.08 (1.95) 0.000**	−0.05	5.86 (2.17) 0.007**	−0.07
Within‐Person Fixed Effects										
Time ($\gamma_{10})$	−0.01 (0.01) 0.072	−0.03	−0.00 (0.01) 0.855	−0.00	0.00 (0.01) 0.850	0.00	0.02 (0.01) 0.014*	0.04	−0.00 (0.01) 0.565	−0.01
Weekday ($\gamma_{20})$	0.21 (0.07) 0.005**	0.10	0.20 (0.08) 0.007**	0.08	0.18 (0.07) 0.015*	0.08	−0.20 (0.08) 0.010*	−0.09	−0.09 (0.08) 0.256	−0.03
Prior‐day Outcome ($\gamma_{30})$	0.27 (0.02) 0.000**	0.27	0.16 (0.02) 0.000**	0.16	0.26 (0.02) 0.000**	0.25	0.03 (0.02) 0.129	0.04	0.24 (0.02) 0.000**	0.24
Daily PBS ($\gamma_{40})$	−0.04 (0.02) 0.037*	−0.04	0.01 (0.02) 0.543	0.01	−0.03 (0.02) 0.096	−0.03	0.04 (0.02) 0.058	0.03	0.03 (0.02) 0.219	0.02
Between‐Person Fixed Effects										
Avg. PBS ($\gamma_{01}$)	−0.12 (0.04) 0.002**	−0.13	−0.08 (0.06) 0.184	−0.07	−0.10 (0.05) 0.039*	−0.10	0.15 (0.06) 0.011*	0.15	0.15 (0.06) 0.021*	0.13
Youth Age ($\gamma_{02}$)	−0.08 (0.08) 0.323	−0.04	0.06 (0.13) 0.649	0.03	−0.02 (0.10) 0.824	−0.01	−0.14 (0.12) 0.233	−0.07	−0.09 (0.13) 0.495	−0.04
Youth Sex ($\gamma_{03}$)	−0.74 (0.18) 0.000**	−0.35	−0.89 (0.28) 0.002**	−0.37	−0.74 (0.23) 0.002**	−0.33	0.49 (0.27) 0.072	0.22	0.47 (0.30) 0.127	0.17
Family Income ($\gamma_{04}$)	−0.05 (0.03) 0.129	−0.06	0.01 (0.05) 0.834	0.01	−0.05 (0.04) 0.199	−0.06	0.09 (0.05) 0.040*	0.12	0.07 (0.05) 0.188	0.07

Random Effects	***Variance (SD)***		***Variance (SD)***		***Variance (SD)***		***Variance (SD)***		***Variance (SD)***	
Intercept ($u_{0i}$)	0.82 (0.91)		2.26 (1.50)		1.42 (1.19)		2.08 (1.44)		2.65 (1.63)	
Daily PBS ($u_{1i}$)	0.00 (0.00)		0.01 (0.08)		0.00 (0.04)		0.01 (0.08)		0.02 (0.13)	
Residual ($e_{\mathrm{it}})$	2.52 (1.59)		2.30 (1.52)		2.21 (1.49)		2.55 (1.60)		2.67 (1.64)	

Abbreviations: Avg., average (between‐person variables); Daily, within‐person variables; Est, estimate; *p*, *p* value; PBS, parents' use of positive behavior support; SE, standard error; β, standardized coefficient.

*p < 0.05; **p < 0.01.

**Figure 1:**
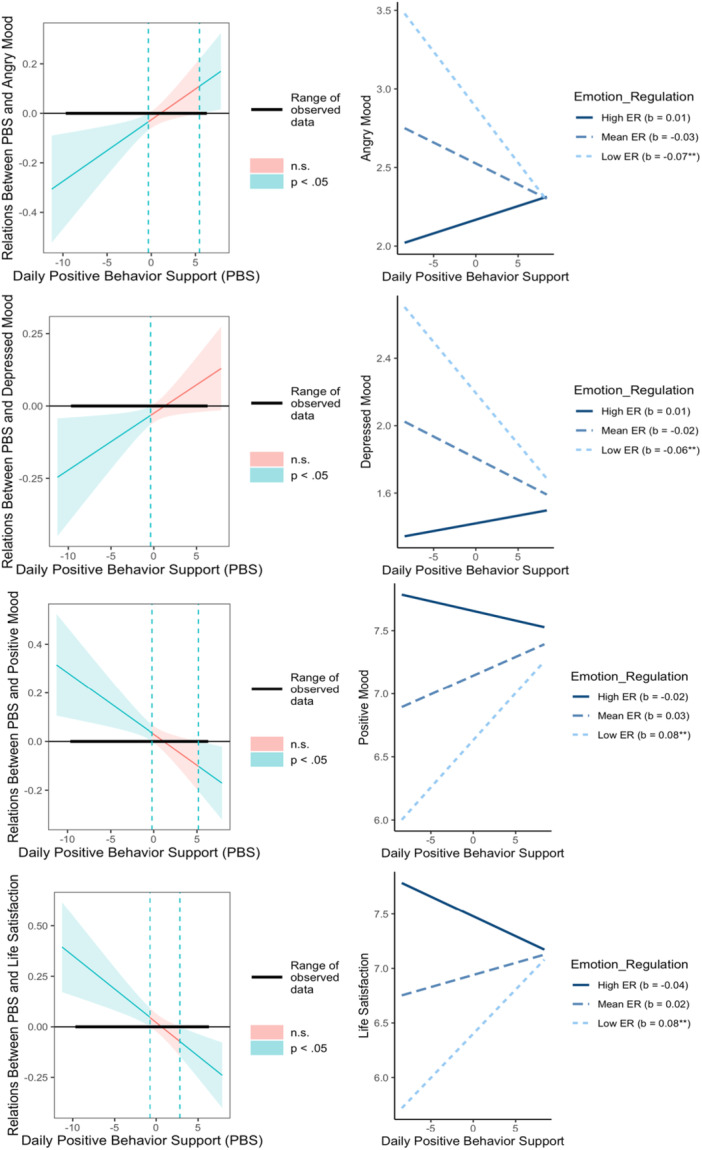
The moderating role of daily adolescent emotion regulation. *Note:* PBS, parent's use of positive behavior support; low ER means 1 SD below the mean of adolescent emotion regulation; high ER means 1 SD above the mean of adolescent emotion regulation. ER, emotion regulation; Est., estimate.

To test the second hypothesis (see Supporting Information: Equation 3‐4 g), we estimated multilevel models evaluating the moderating role of adolescent emotion regulation in the daily association between parents' use of positive behavior support and adolescent well‐being. Consistent with the support needs perspective (see Table 4), results revealed four statistically significant interaction effects for angry (*b* = 0.02, *p* < 0.05), depressed (*b* = 0.02, *p* < 0.05), positive mood (*b* = −0.03, *p* < 0.01), and life satisfaction (*b* = −0.03, *p* < 0.01). The interaction effect for anxious mood was not significant (*b* = 0.01, *p* = 0.176). We probed the interactions using simple slope and Johnson‐Neyman analyses (see Figure 1). Johnson‐Neyman results indicated that daily positive behavior support was associated with better adolescent well‐being on days when adolescents reported lower‐than‐usual emotion regulation (i.e., below −0.33 for angry mood, −0.37 for depressed mood, −0.20 for positive mood, and −0.75 for life satisfaction). At the other end of the adolescent emotion regulation continuum, daily positive behavior support was associated with poorer well‐being for angry mood (> 5.46; 2 observations), positive mood (> 5.16; 7 observations), and life satisfaction (> 2.83; 98 observations); however, the regions of significance were based on a limited number of observations relative to the total number of daily reports. Both positive behavior support and emotion regulation were person‐mean centered; therefore, interaction terms and Johnson‐Neyman values reflect daily deviations from each participant's typical levels.

**Table 4:** Testing the role of daily adolescent emotion regulation in parents' use of positive behavior support and adolescent well‐being.

	Angry Mood		Anxious Mood		Depressed Mood		Positive Mood		Life Satisfaction	
	***B (SE) p***	β	***Est (SE) p***	β	***Est (SE) p***	β	***Est (SE) p***	β	***Est (SE) p***	β
Intercept ($\gamma_{00}$)	3.84 (1.24) 0.002**	0.10	2.29 (2.00) 0.253	0.10	3.27 (1.56) 0.036*	0.08	7.46 (1.44) 0.000**	−0.01	6.63 (1.74) 0.000**	−0.04
Within‐Person Fixed Effects										
Time ($\gamma_{10})$	−0.01 (0.01) 0.021*	−0.04	−0.00 (0.01) 0.598	−0.01	−0.00 (0.01) 0.898	−0.00	0.02 (0.01) 0.003**	0.05	−0.00 (0.01) 0.618	−0.01
Weekday ($\gamma_{20})$	0.21 (0.07) 0.004**	0.10	0.22 (0.07) 0.002**	0.09	0.18 (0.07) 0.014*	0.08	−0.20 (0.07) 0.007**	−0.09	−0.11 (0.07) 0.146	−0.04
Prior‐day Outcome ($\gamma_{30})$	0.23 (0.02) 0.000**	0.23	0.05 (0.02) 0.014*	0.05	0.16 (0.02) 0.000**	0.16	0.05 (0.02) 0.009**	0.06	0.18 (0.02) 0.000**	0.18
Daily PBS ($\gamma_{40})$	−0.03 (0.02) 0.083	−0.03	0.02 (0.02) 0.370	0.01	−0.02 (0.02) 0.128	−0.02	0.03 (0.02) 0.120	0.03	0.02 (0.02) 0.367	0.02
Daily ER ($\gamma_{50})$	−0.20 (0.03) 0.000**	−0.17	−0.20 (0.03) 0.000**	−0.14	−0.22 (0.03) 0.000**	−0.17	0.29 (0.03) 0.000**	0.23	0.30 (0.03) 0.000**	0.20
Daily PBS * Daily ER ($\gamma_{60})$	0.02 (0.01) 0.015*	0.04	0.01 (0.01) 0.176	0.02	0.02 (0.01) 0.031*	0.03	−0.03 (0.01) 0.006**	−0.04	−0.03 (0.01) 0.001**	−0.05
Between‐Person Fixed Effects										
Avg. PBS ($\gamma_{01}$)	−0.08 (0.04) 0.026*	−0.09	−0.05 (0.06) 0.409	−0.05	−0.05 (0.05) 0.291	−0.05	0.08 (0.04) 0.081	0.08	0.08 (0.05) 0.134	0.07
Avg. ER ($\gamma_{02}$)	−0.19 (0.05) 0.000**	−0.17	−0.43 (0.08) 0.000**	−0.33	−0.36 (0.06) 0.000**	−0.30	0.45 (0.06) 0.000**	0.37	0.61 (0.07) 0.000**	0.42
Youth Age ($\gamma_{03}$)	−0.10 (0.08) 0.176	−0.06	−0.01 (0.12) 0.926	−0.01	−0.09 (0.10) 0.370	−0.04	−0.09 (0.09) 0.288	−0.05	−0.07 (0.11) 0.521	−0.03
Youth Sex ($\gamma_{04}$)	−0.74 (0.17) 0.000**	−0.35	−0.98 (0.28) 0.001**	−0.40	−0.70 (0.22) 0.002**	−0.31	0.30 (0.20) 0.139	0.13	0.35 (0.24) 0.150	0.13
Family Income ($\gamma_{05}$)	−0.02 (0.03) 0.399	−0.03	0.02 (0.05) 0.619	0.03	−0.03 (0.04) 0.337	−0.05	0.07 (0.03) 0.032*	0.10	0.01 (0.04) 0.814	0.01

Random Effects	***Variance (SD)***		***Variance (SD)***		***Variance (SD)***		***Variance (SD)***		***Variance (SD)***	
Intercept ($u_{0i}$)	0.80 (0.90)		2.25 (1.50)		1.40 (1.18)		1.22 (1.10)		1.69 (1.30)	
Daily PBS ($u_{1i}$)	0.00 (0.05)		0.00 (0.05)		0.00 (0.04)		0.01 (0.07)		0.02 (0.14)	
Daily ER ($u_{2i}$)	0.03 (0.17)		0.07 (0.26)		0.05 (0.21)		0.04 (0.20)		0.06 (0.25)	
Residual ($e_{\mathrm{it}})$	2.30 (1.52)		2.07 (1.44)		1.97 (1.40)		2.15 (1.47)		2.17 (1.47)	

Abbreviations: Avg., average (between‐person variables); Daily, within‐person variables; Est, estimate; *p*, *p* value; PBS, parents' use of positive behavior support; SE, standard error; β, standardized coefficient.

*p < 0.05; **p < 0.01.

It is worth noting that both daily and average levels of emotion regulation showed significant associations with adolescent well‐being across all models. On days when emotion regulation was higher than usual, adolescents experienced lower negative moods (*bs* = −0.22 to −0.20, *ps* < 0.01) and higher positive mood and life satisfaction (*bs* = 0.29 to 0.30, *ps* < 0.01). In addition, adolescents with higher average levels of emotion regulation reported lower negative mood (*bs* = −0.43 to −0.19, *ps* < 0.01) and higher positive mood and life satisfaction (*bs* = 0.45 to 0.61, *ps* < 0.01).

In post hoc analyses (see Supporting Information: Equation 5‐6 f), we examined whether between‐person differences in average adolescent emotion regulation moderated the daily association between parents' use of positive behavior support and adolescent well‐being. Cross‐level moderation analyses revealed no statistically significant moderation effects in any of the models (all *ps* > 0.60; see Table S1). As a sensitivity analysis, we applied FDR adjustments to account for multiple comparisons across models. Results revealed similar patterns (see Tables S2–S4), with the changes being that daily positive behavior support was no longer significantly associated with adolescent angry mood (adjusted *p* = 0.055), and the moderating role of daily adolescent emotion regulation in the association between positive behavior support and adolescent depressed mood was also no longer significant (adjusted *p* = 0.061).

## 4 Discussion

Building on recent work highlighting within‐person fluctuations in positive behavior support parenting, adolescent well‐being, and emotion regulation (e.g., Blanke et al. 2020; Chen et al. 2025; Fosco and Lydon‐Staley 2020), this study was designed to evaluate whether daily fluctuations in positive behavior support explained changes in adolescent daily well‐being, and whether such relations were qualified by day‐to‐day changes in adolescents' sense of emotion regulation.

### 4.1 Daily Parents' Use of Positive Behavior Support and Adolescent Well‐Being

Our first set of analyses evaluated whether daily parents' use of positive behavior support was linked to adolescent well‐being. Our findings extend prior research demonstrating the benefits of positive behavior support in children's development (e.g., Dishion et al. 2008) to adolescence, suggesting that positive behavior support remains an effective parenting strategy across developmental periods. As expected, at the between‐person level, parents who used more positive behavior support on average had adolescents who reported lower angry and depressed mood, as well as higher positive mood and life satisfaction.

Drawing on the advantages of daily diary data, we distinguished between‐person and within‐person differences in positive behavior support parenting (Bamberger 2016; Laurenceau and Bolger 2005); for instance, parents who on average report higher positive behavior support still show ups and downs across days. Interestingly, at the within‐person level, when accounting for prior‐day well‐being, daily fluctuations in positive behavior support did not show robust associations with changes in adolescent well‐being. Although the unadjusted models suggested that days with higher‐than‐usual positive behavior support were associated with lower angry mood, this association was not significant after FDR adjustment and was therefore not sufficiently robust to draw firm conclusions. Our findings support the notion that parenting processes may function differently across timescales (Boele et al. 2020). Parents who consistently use positive behavior support over time may create a cumulative pattern of reinforcement that shapes adolescents' overall well‐being, whereas the effects of any single day's parenting behavior may be too subtle to detect above and beyond prior‐day well‐being. However, these findings may be deceiving by not accounting for changes in within‐person states that may affect the degree to which daily positive behavior support is linked with daily variation in adolescent well‐being.

### 4.2 Daily Adolescent Emotion Regulation as a Moderator

The second aim of this study was to evaluate whether daily fluctuations in adolescent emotion regulation moderated the within‐person association between positive behavior support parenting and adolescent well‐being. We considered two possibilities: a resource amplification perspective posits that higher daily emotion regulation would amplify the benefits of parental support by providing greater cognitive resources to recognize and utilize it; alternatively, a support needs perspective suggests that on days when adolescents experience poorer emotion regulation, they may be more receptive to parental support. Our findings supported the support needs perspective. On days when adolescents reported lower emotion regulation than usual, daily positive behavior support was significantly associated with better well‐being, as indicated by lower angry and depressed mood as well as higher positive mood and life satisfaction. However, on days when adolescents reported higher emotion regulation than usual, daily positive behavior support was associated with higher angry mood, lower positive mood, and lower life satisfaction, though these regions of significance were based on limited observations and should be interpreted with caution.

These findings align with research demonstrating that emotion regulation is inherently dynamic and characterized by meaningful daily fluctuations (Blanke et al. 2020; Silk et al. 2003; Vanderlind et al. 2022). Within the support needs framework, daily changes in adolescents' sense of emotion regulation may alter how they interpret and respond to the same positive behavior support across days (Soenens et al. 2015). On days when adolescents experience poorer emotion regulation, they may seek or elicit greater parental support and emotional validation to help manage their emotions. Additionally, on days when adolescents report lower emotion regulation, they may also experience greater emotional distress, providing more room for improvement in response to positive parenting compared with days when well‐being is already high. Conversely, considering adolescents' developmental needs for autonomy (Soenens et al. 2007), on days when adolescents are coping effectively, they may function more autonomously and perceive increased positive behavior support as unnecessary or intrusive.

Follow‐up analyses revealed that average adolescent emotion regulation did not significantly moderate the daily association between positive behavior support and well‐being. Notably, the random slopes for daily positive behavior support were also non‐significant across all models, indicating that the daily association between positive behavior support and well‐being operates similarly across adolescents. Accordingly, the current sample does not support strong claims about stable between‐person heterogeneity in these daily associations. Instead, it is day‐to‐day fluctuations in emotion regulation within a given adolescent that meaningfully explain when positive behavior support is most beneficial. These findings contribute to parenting theory by disentangling within‐person and between‐person processes to examine ideas of heterogeneity in parenting effects—a central feature of contemporary models (e.g., Boele et al. 2020).

Notably, adolescent emotion regulation demonstrated robust associations with well‐being at both between‐ and within‐person levels, even when accounting for daily positive parenting. It is possible that this finding may be inflated due to shared‐reporter variance (emotion regulation and well‐being were both reported by adolescents), whereas positive behavior support was reported by parents, which may have resulted in a weaker statistical relation. However, our findings are consistent with emotion regulation theory (Gross 2015), effective emotion regulation enables adolescents to modulate emotional experiences in ways that facilitate adaptive functioning and stress management. Viewed through the support needs perspective, emotion regulation may largely sustain adolescent well‐being on its own; but on days when adolescents' regulatory capacity is diminished, parents' use of positive behavior support may serve as an important external resource that bolsters well‐being.

Interestingly, our results did not reveal a significant association between daily positive behavior support and adolescent anxious mood regardless of emotion regulation. This aligns with meta‐analytic evidence indicating that positive parenting plays a limited role in predicting adolescents' anxiety; but parental autonomy support accounts for substantially more variance (McLeod et al. 2007). Because anxiety is fundamentally tied to a perceived lack of control and anticipation of future threat, what matters most for anxiety is whether parents foster adolescents' sense of autonomy and confidence in handling uncertainty (McLeod et al. 2007). Positive behavior support does not directly address adolescents' perceived competence to cope independently with challenges, which are the mechanisms most relevant to anxiety. In contrast, positive parenting is strongly related to lower depression in youth (McLeod et al. 2007), suggesting that different dimensions of negative mood have distinct associations with parents' use of positive behavior support.

### 4.3 Limitations and Future Directions

Although the current study offers significant insights, a few limitations should be acknowledged. First, the sample primarily consisted of White adolescents from higher socioeconomic backgrounds. Future work would benefit from including more diverse populations to generalize the findings. Second, emotion regulation and well‐being were both assessed via adolescent self‐report, whereas positive behavior support was reported by parents. Future research should incorporate adolescent reports of parenting to better disentangle substantive associations from reporter effects. Third, although the current study captures meaningful concurrent associations between parents' use of positive behavior support and adolescent well‐being, the cross‐sectional design limits conclusions about temporal ordering and bidirectional processes. Future work collecting data at multiple occasions within each day could provide richer insights into the dynamic nature of these associations. Fourth, daily surveys asked participants to report on the previous day's experiences after a night of sleep, which may have introduced greater recall bias compared with typical end‐of‐day diary designs. Finally, the use of single‐item and two‐item measures may not fully capture the complexity of the constructs; future studies would benefit from using more comprehensive multi‐item assessments.

### 4.4 Implications

The current findings yield several implications. First, we extended evidence on parenting effect heterogeneity to the daily timescale, identifying within‐person emotion regulation as a key factor that meaningfully explains within‐person variability in the association between positive behavior support and adolescent well‐being. Second, our results extend prior findings on the benefits of positive behavior support from childhood (e.g., Dishion et al. 2008) to adolescence, suggesting that this parenting strategy continues to play a meaningful role in adolescent well‐being. Third, the significance of between‐person rather than within‐person effects of positive behavior support highlights the importance of disentangling these levels of analysis in parenting research. Fourth, disaggregating adolescent negative mood revealed that different mood dimensions function uniquely in the parenting context. Fifth, adolescent emotion regulation emerged as a robust correlate of well‐being at both within‐ and between‐person levels, even when accounting for daily parenting. From a practical perspective, these findings suggest that family‐based intervention efforts may benefit from incorporating attention to daily changes in adolescent emotion regulation to help parents provide timely, developmentally appropriate support.

## Author Contributions

**Lan Chen:** conceptualization, software, formal analysis, funding acquisition, writing – original draft, writing – review and editing, visualization, data curation. **Gregory M. Fosco:** conceptualization, methodology, funding acquisition, writing – review and editing, project administration, supervision, resources, validation, investigation.

## Ethics Statement

All procedures performed in this study involving human subjects were in accordance with the ethical standards of the institutional and/or national research committee and with the 1964 Helsinki Declaration and its later amendments or comparable ethical standards. All procedures performed in the study involving human participants were approved by the Institutional Review Board at the Penn State University Institutional Review Board (IRB protocol number: 00015632).

## Consent

Informed consent/assent was obtained from all participants included in the study.

## Conflicts of Interest

The authors declare no conflicts of interest.

## Supporting information

Supporting File

## Data Availability

The data that support the findings of this study are available on request from the corresponding author. The data are not publicly available due to privacy or ethical restrictions.
