# Supplementary material for: Dynamic Links Between Daily Positive Parenting and Adolescent Well‐Being: The Moderating Role of Daily Adolescent Emotion Regulation
Source: J Adolesc. 2026 May 14;98(5):1767–79. doi: 10.1002/jad.70179 (PMC13338661; doi:10.1002/jad.70179)
Supplement: Supplementary file 1 — Supporting File [file JAD-98-1767-s001.docx]

**S1.** *Model Specifications and Formulas*

**Hypothesis1**. To test the daily association between parents’ use of positive behavior support and adolescent well-being, the model is specified as follows:

At level 1 (day-level variables), the equation is constructed as:

$$\left( 1 \right){AWellbeing}_{it}=\beta_{0i}+{\beta_{1i}{Time}_{it}+\beta_{2i}{Weekday}_{it}+\beta_{3i}{Wellbeing}_{it-1}+\beta}_{4i}Day^{'}s{PBS}_{it}+e_{it}$$

where ${AWellbeing}_{it}$ reflects the adolescent each aspect of well-being for individual *i* on day *t*; $\beta_{0i}$ reflects the expected adolescent well-being in the middle of the study for an individual experiencing an average level of daily parents’ use of positive behavior support; $\beta_{1i}$ indicates the association between time in the study and daily adolescent well-being; $\beta_{2i}$ indicates the relations between weekday and adolescent well-being; $\beta_{3i}$ indicates the relations between prior-day well-being and adolescent well-being; $\beta_{4i}$ indicates the relations between parents’ use of positive behavior support and adolescent well-being. Finally, $e_{it}$ are day-specific residuals, which were allowed to be autocorrelated following a first-order autoregressive structure (AR(1)).

At the level 2 (individual-level variables), person-specific intercepts and slopes from the Level 1 model are specified as:

$$\left( 2a \right)\beta_{0i}=\gamma_{00}+\gamma_{01}{UsualPBS}_{i}{{{+\gamma}_{02}{Age}_{i}+\gamma}_{03}{Sex}_{i}+\gamma}_{04}{FIncome}_{i}+u_{0i}$$

$$\left( 2b \right)\beta_{1i}=\gamma_{10}$$

$$\left( 2c \right)\beta_{2i}=\gamma_{20}$$

$$\left( 2d \right)\beta_{3i}=\gamma_{30}$$

$$\left( 2e \right)\beta_{4i}=\gamma_{40}+u_{1i}$$

Here, the $\gamma$s are sample-level parameters, and the $u$s are residual individual differences. These equations show the association between between-person parents’ use of positive behavior support, adolescent age, adolescent sex, and family income with adolescent well-being, as represented by $\gamma_{01}$, $\gamma_{02},$ $\gamma_{03}$, and $\gamma_{04}$ respectively. $u_{0i}$ and $u_{1i}$ represent the random effects.

**Hypothesis 2.** To test the moderating role of daily adolescent emotion regulation in shaping the daily linkage between parents’ use of positive behavior support and adolescent well-being, we incorporated an interaction term for the within-person coefficients in our multilevel models. We implemented the second set of multilevel models:

$${\left( 3 \right) AWellbeing}_{it}=\beta_{0i}+{\beta_{1i}{Time}_{it}+\beta_{2i}{Weekday}_{it}+\beta_{3i}{Wellbeing}_{it-1}+\beta}_{4i}Day^{'}s{PBS}_{it}+{\beta_{5i}{Day^{'}sER}_{it}+ \beta_{6i}{Day^{'}sPBS}_{it} X{Day^{'}sER}_{it}+e}_{it}$$

$${\left( 4a \right) \beta}_{0i}= \gamma_{00}+\gamma_{01}{UsualPBS}_{i}{{{{+\gamma}_{02}{UsualER}_{i}+\gamma}_{03}{Age}_{i}+\gamma}_{04}{Sex}_{i}+\gamma}_{05}{FIncome}_{i}+u_{0i}$$

$$\left( 4b \right)\beta_{1i}=\gamma_{10}$$

$$\left( 4c \right)\beta_{2i}=\gamma_{20}$$

$$\left( 4d \right)\beta_{3i}=\gamma_{30}$$

$$\left( 4e \right)\beta_{4i}=\gamma_{40}+u_{1i}$$

$$\left( 4d \right)\beta_{5i}=\gamma_{50}+u_{2i}$$

$$\left( 4g \right)\beta_{6i}=\gamma_{60}$$

In these equations, $\gamma$s represent sample-level fixed effects, and $u$s represent residual individual differences (random effects).

**Post-Hoc Analysis.** To test the moderating role of general levels of adolescent emotion regulation in shaping the daily linkage between parents’ use of positive behavior support and adolescent well-being, we incorporated an cross-level interaction term in our multilevel models. We implemented the second set of multilevel models:

$${\left( 5 \right) AWellbeing}_{it}=\beta_{0i}+{\beta_{1i}{Time}_{it}+\beta_{2i}{Weekday}_{it}+\beta_{3i}{Wellbeing}_{it-1}+\beta}_{4i}Day^{'}s{PBS}_{it}+{\beta_{5i}{Day^{'}sER}_{it}+e}_{it}$$

$${\left( 6a \right) \beta}_{0i}= \gamma_{00}+\gamma_{01}{UsualPBS}_{i}{{{{+\gamma}_{02}{UsualER}_{i}+\gamma}_{03}{Age}_{i}+\gamma}_{04}{Sex}_{i}+\gamma}_{05}{FIncome}_{i}+u_{0i}$$

$$\left( 6b \right)\beta_{1i}=\gamma_{10}$$

$$\left( 6c \right)\beta_{2i}=\gamma_{20}$$

$$\left( 6d \right)\beta_{3i}=\gamma_{30}$$

$$\left( 6e \right)\beta_{4i}=\gamma_{40}+{\gamma_{41}{UsualER}_{i}+u}_{1i}$$

$$\left( 6f \right)\beta_{5i}=\gamma_{50}+u_{2i}$$

In these equations, $\gamma$s represent sample-level fixed effects, and $u$s represent residual individual differences (random effects).

**Table S1.** *Testing the moderating role of average adolescent emotion regulation in the association between daily parents’ use of positive behavior support and adolescent well-being.*

|  | Angry Mood | | | Anxious Mood | | Depressed Mood | | Positive Mood | | | Life Satisfaction | |
| --- | --- | --- | --- | --- | --- | --- | --- | --- | --- | --- | --- | --- |
| Main effect | ***B (SE) p*** | $\boldsymbol{\beta}$ | ***Est (SE) p*** | | $\boldsymbol{\beta}$ | ***Est (SE) p*** | $\boldsymbol{\beta}$ | | ***Est (SE) p*** | $\boldsymbol{\beta}$ | ***Est (SE) p*** | $\boldsymbol{\beta}$ |
| Intercept ($\boldsymbol{\gamma}_{\boldsymbol{00}}$) | 3.79 (1.24) .002** | 0.09 | 2.31 (2.00) .248 | | 0.10 | 3.33 (1.57) .034* | 0.08 | | 7.45 (1.43) .000** | -0.00 | 6.67 (1.76) .000** | -0.04 |
| *Within-Person Fixed Effects* |  |  |  | |  |  |  | |  |  |  |  |
| Time ($\boldsymbol{\gamma}_{\boldsymbol{10}}\boldsymbol{)}$ | -0.01 (0.01) .021* | -0.04 | -0.00 (0.01) .590 | | -0.01 | -0.00 (0.01) .887 | -0.00 | | 0.02 (0.01) .002** | 0.05 | -0.00 (0.01) .641 | -0.01 |
| Weekday ($\boldsymbol{\gamma}_{\boldsymbol{20}}\boldsymbol{)}$ | 0.21 (0.07) .004** | 0.10 | 0.23 (0.07) .002** | | 0.09 | 0.18 (0.07) .013* | 0.08 | | -0.20 (0.07) .007** | -0.09 | -0.11 (0.07) .134 | -0.04 |
| Prior-day Outcome ($\boldsymbol{\gamma}_{\boldsymbol{30}}\boldsymbol{)}$ | 0.24 (0.02) .000** | 0.24 | 0.05 (0.02) .015* | | 0.05 | 0.15 (0.02) .000** | 0.16 | | 0.05 (0.02) .009** | 0.06 | 0.18 (0.02) .000** | 0.17 |
| Daily PBS ($\boldsymbol{\gamma}_{\boldsymbol{40}}\boldsymbol{)}$ | -0.03 (0.02) .093 | -0.03 | 0.02 (0.02) .370 | | 0.01 | -0.02 (0.02) .128 | -0.02 | | 0.03 (0.02) .090 | 0.03 | 0.02 (0.02) .282 | 0.02 |
| Daily ER ($\boldsymbol{\gamma}_{\boldsymbol{50}}\boldsymbol{)}$ | -0.20 (0.03) .000** | -0.17 | -0.20 (0.03) .000** | | -0.15 | -0.22 (0.03) .000** | -0.17 | | 0.29 (0.03) .000** | 0.23 | 0.31 (0.03) .000** | 0.20 |
| *Between-Person Fixed Effects* |  |  |  | |  |  |  | |  |  |  |  |
| Avg. PBS ($\boldsymbol{\gamma}_{\boldsymbol{01}}$) | -0.08 (0.04) .029* | -0.09 | -0.05 (0.06) .417 | | -0.05 | -0.05 (0.05) .324 | -0.05 | | 0.07 (0.04) .089 | 0.07 | 0.08 (0.05) .146 | 0.06 |
| Avg. ER ($\boldsymbol{\gamma}_{\boldsymbol{02}}$) | -0.19 (0.05) .000** | -0.17 | -0.44 (0.08) .000** | | -0.34 | -0.36 (0.06) .000** | -0.30 | | 0.45 (0.06) .000** | 0.37 | 0.61 (0.07) .000** | 0.42 |
| Youth Age ($\boldsymbol{\gamma}_{\boldsymbol{03}}$) | -0.10 (0.08) .182 | -0.05 | -0.01 (0.12) .917 | | -0.01 | -0.09 (0.10) .350 | -0.04 | | -0.09 (0.09) .293 | -0.05 | -0.07 (0.11) .526 | -0.03 |
| Youth Sex ($\boldsymbol{\gamma}_{\boldsymbol{04}}$) | -0.73 (0.17) .000** | -0.34 | -0.98 (0.28) .001** | | -0.40 | -0.70 (0.22) .002** | -0.31 | | 0.29 (0.20) .155 | 0.13 | 0.34 (0.25) .170 | 0.13 |
| Family Income ($\boldsymbol{\gamma}_{\boldsymbol{05}}$) | -0.02 (0.03) .420 | -0.03 | 0.02 (0.05) .621 | | 0.03 | -0.03 (0.04) .353 | -0.04 | | 0.07 (0.03) .033* | 0.09 | 0.01 (0.04) .819 | 0.01 |
| *Cross-Level Interactions* |  |  |  | |  |  |  | |  |  |  |  |
| Daily PBS * Avg. ER ($\boldsymbol{\gamma}_{\boldsymbol{41}}\boldsymbol{)}$ | 0.00 (0.01) .848 | 0.00 | -0.01 (0.01) .599 | | -0.01 | 0.00 (0.01) .936 | 0.00 | | 0.00 (0.01) .985 | 0.00 | -0.00 (0.01) .737 | -0.01 |
| Random Effects | ***Variance (SD)*** | | | ***Variance (SD)*** | | ***Variance (SD)*** | | ***Variance (SD)*** | | | ***Variance (SD)*** | |
| Intercept ($\boldsymbol{u}_{\boldsymbol{0}\boldsymbol{i}}$) | 0.80 (0.90) | | | 2.25 (1.50) | | 1.42 (1.19) | | 1.23 (1.11) | | | 1.73 (1.32) | |
| Daily PBS ($\boldsymbol{u}_{\boldsymbol{1}\boldsymbol{i}}$) | 0.00 (0.05) | | | 0.00 (0.05) | | 0.00 (0.04) | | 0.01 (0.07) | | | 0.02 (0.14) | |
| Daily ER ($\boldsymbol{u}_{\boldsymbol{2}\boldsymbol{i}}$) | 0.03 (0.17) | | | 0.07 (0.26) | | 0.04 (0.21) | | 0.04 (0.21) | | | 0.06 (0.25) | |
| Residual ($\boldsymbol{e}_{\boldsymbol{it}}\boldsymbol{)}$ | 2.30 (1.52) | | | 2.07 (1.44) | | 1.97 (1.40) | | 2.16 (1.47) | | | 2.18 (1.48) | |

***Note.*** Est = estimate; SE = standard error; p = p value; β = standardized coefficient; PBS = parents’ use of positive behavior support; Avg. = average (between-person variables); ER = emotion regulation; Daily = within-person variables. * indicates *p* < .05. ** indicates *p* < .01.

**Table S2.** *False discovery rate (FDR)-adjusted p-values for multilevel models examining associations between daily positive behavior support and adolescent well-being outcomes.*

|  | Angry Mood | *Anxious Mood* | *Depressed Mood* | *Positive Mood* | *Life Satisfaction* |
| --- | --- | --- | --- | --- | --- |
|  | ***Adjusted P-value*** | ***Adjusted P-value*** | ***Adjusted P-value*** | ***Adjusted P-value*** | ***Adjusted P-value*** |
| Intercept ($\boldsymbol{\gamma}_{\boldsymbol{00}}$) | 0.010 | 0.835 | 0.254 | 0.000 | 0.031 |
| *Within-Person Fixed Effects* |  |  |  |  |  |
| Time ($\boldsymbol{\gamma}_{\boldsymbol{10}}\boldsymbol{)}$ | 0.092 | 0.855 | 0.850 | 0.032 | 0.565 |
| Weekday ($\boldsymbol{\gamma}_{\boldsymbol{20}}\boldsymbol{)}$ | 0.010 | 0.022 | 0.045 | 0.032 | 0.329 |
| Prior-day Outcome ($\boldsymbol{\gamma}_{\boldsymbol{30}}\boldsymbol{)}$ | 0.000 | 0.000 | 0.000 | 0.146 | 0.000 |
| Daily PBS ($\boldsymbol{\gamma}_{\boldsymbol{40}}\boldsymbol{)}$ | 0.055 | 0.835 | 0.173 | 0.086 | 0.328 |
| *Between-Person Fixed Effects* |  |  |  |  |  |
| Avg. PBS ($\boldsymbol{\gamma}_{\boldsymbol{01}}$) | 0.007 | 0.413 | 0.087 | 0.032 | 0.064 |
| Youth Age ($\boldsymbol{\gamma}_{\boldsymbol{02}}$) | 0.323 | 0.835 | 0.850 | 0.233 | 0.557 |
| Youth Sex ($\boldsymbol{\gamma}_{\boldsymbol{03}}$) | 0.000 | 0.010 | 0.007 | 0.093 | 0.287 |
| Family Income ($\boldsymbol{\gamma}_{\boldsymbol{04}}$) | 0.146 | 0.855 | 0.255 | 0.071 | 0.328 |

***Note*:** Est = estimate; SE = standard error; p = p value; PBS = parents’ use of positive behavior support; Avg. = average (between-person variables); Corr = correlation; Daily = within-person variables.

**Table S3.** *False discovery rate (FDR)-adjusted p-values for multilevel models examining the moderating role of daily adolescent emotion regulation in the association between daily parents’ use of positive behavior support and adolescent well-being.*

|  | Angry Mood | *Anxious Mood* | *Depressed Mood* | *Positive Mood* | *Life Satisfaction* |
| --- | --- | --- | --- | --- | --- |
|  | ***Adjusted P-value*** | ***Adjusted P-value*** | ***Adjusted P-value*** | ***Adjusted P-value*** | ***Adjusted P-value*** |
| Intercept ($\boldsymbol{\gamma}_{\boldsymbol{00}}$) | 0.005 | 0.433 | 0.062 | 0.000 | 0.000 |
| *Within-Person Fixed Effects* |  |  |  |  |  |
| Time ($\boldsymbol{\gamma}_{\boldsymbol{10}}\boldsymbol{)}$ | 0.032 | 0.675 | 0.898 | 0.008 | 0.674 |
| Weekday ($\boldsymbol{\gamma}_{\boldsymbol{20}}\boldsymbol{)}$ | 0.008 | 0.007 | 0.033 | 0.015 | 0.225 |
| Prior-day Outcome ($\boldsymbol{\gamma}_{\boldsymbol{30}}\boldsymbol{)}$ | 0.000 | 0.034 | 0.000 | 0.015 | 0.000 |
| Daily PBS ($\boldsymbol{\gamma}_{\boldsymbol{40}}\boldsymbol{)}$ | 0.099 | 0.545 | 0.192 | 0.143 | 0.489 |
| Daily ER ($\boldsymbol{\gamma}_{\boldsymbol{50}}\boldsymbol{)}$ | 0.000 | 0.000 | 0.000 | 0.000 | 0.000 |
| Daily PBS * Daily ER ($\boldsymbol{\gamma}_{\boldsymbol{60}}\boldsymbol{)}$ | 0.026 | 0.351 | 0.061 | 0.014 | 0.002 |
| *Between-Person Fixed Effects* |  |  |  |  |  |
| Avg. PBS ($\boldsymbol{\gamma}_{\boldsymbol{01}}$) | 0.035 | 0.545 | 0.388 | 0.108 | 0.225 |
| Avg. ER ($\boldsymbol{\gamma}_{\boldsymbol{02}}$) | 0.000 | 0.000 | 0.000 | 0.000 | 0.000 |
| Youth Age ($\boldsymbol{\gamma}_{\boldsymbol{03}}$) | 0.192 | 0.926 | 0.404 | 0.287 | 0.626 |
| Youth Sex ($\boldsymbol{\gamma}_{\boldsymbol{04}}$) | 0.000 | 0.003 | 0.005 | 0.151 | 0.225 |
| Family Income ($\boldsymbol{\gamma}_{\boldsymbol{05}}$) | 0.399 | 0.675 | 0.404 | 0.047 | 0.814 |

***Note.*** Est = estimate; SE = standard error; p = p value; PBS = parents’ use of positive behavior support; Avg. = average; ER = emotion regulation; Corr = correlation.

**Table S4.** *False discovery rate (FDR)-adjusted p-values for multilevel models examining the moderating role of average adolescent emotion regulation in the association between daily parents’ use of positive behavior support and adolescent well-being.*

|  | Angry Mood | *Anxious Mood* | *Depressed Mood* | *Positive Mood* | *Life Satisfaction* |
| --- | --- | --- | --- | --- | --- |
|  | ***Adjusted P-value*** | ***Adjusted P-value*** | ***Adjusted P-value*** | ***Adjusted P-value*** | ***Adjusted P-value*** |
| Intercept ($\boldsymbol{\gamma}_{\boldsymbol{00}}$) | 0.006 | 0.496 | 0.067 | 0.000 | 0.000 |
| *Within-Person Fixed Effects* |  |  |  |  |  |
| Time ($\boldsymbol{\gamma}_{\boldsymbol{10}}\boldsymbol{)}$ | 0.036 | 0.678 | 0.936 | 0.007 | 0.769 |
| Weekday ($\boldsymbol{\gamma}_{\boldsymbol{20}}\boldsymbol{)}$ | 0.008 | 0.006 | 0.030 | 0.017 | 0.291 |
| Prior-day Outcome ($\boldsymbol{\gamma}_{\boldsymbol{30}}\boldsymbol{)}$ | 0.000 | 0.035 | 0.000 | 0.017 | 0.000 |
| Daily PBS ($\boldsymbol{\gamma}_{\boldsymbol{40}}\boldsymbol{)}$ | 0.124 | 0.625 | 0.219 | 0.120 | 0.423 |
| Daily ER ($\boldsymbol{\gamma}_{\boldsymbol{50}}\boldsymbol{)}$ | 0.000 | 0.000 | 0.000 | 0.000 | 0.000 |
| *Between-Person Fixed Effects* |  |  |  |  |  |
| Avg. PBS ($\boldsymbol{\gamma}_{\boldsymbol{01}}$) | 0.043 | 0.625 | 0.424 | 0.120 | 0.291 |
| Avg. ER ($\boldsymbol{\gamma}_{\boldsymbol{02}}$) | 0.000 | 0.000 | 0.000 | 0.000 | 0.000 |
| Youth Age ($\boldsymbol{\gamma}_{\boldsymbol{03}}$) | 0.219 | 0.917 | 0.424 | 0.319 | 0.701 |
| Youth Sex ($\boldsymbol{\gamma}_{\boldsymbol{04}}$) | 0.000 | 0.003 | 0.005 | 0.186 | 0.291 |
| Family Income ($\boldsymbol{\gamma}_{\boldsymbol{05}}$) | 0.458 | 0.678 | 0.424 | 0.057 | 0.819 |
| *Cross-Level Interactions* |  |  |  |  |  |
| Daily PBS * Avg. ER ($\boldsymbol{\gamma}_{\boldsymbol{41}}\boldsymbol{)}$ | 0.848 | 0.678 | 0.936 | 0.985 | 0.804 |

***Note.*** Est = estimate; SE = standard error; p = p value; PBS = parents’ use of positive behavior support; Avg. = average (between-person variables); ER = emotion regulation; Corr = correlation; Daily = within-person variables.

**S2.** R Code

# Load Packages

```{r}

library(foreign)

library(psych)

library(nlme)

library(rmcorr)

library(Hmisc)

library(lme4)

library(reghelper)

library(effectsize)

library(tidyverse)

```

# Reading in the Data

```{r, warning=FALSE}

yday <- read.spss("ERA Youth Daily Scoring_06.16.2025.sav", to.data.frame = T,use.value.labels = FALSE)

pday <- read.spss("ERA Parent Daily Scoring_135.sav", to.data.frame = T,use.value.labels = FALSE)

pday$ID <- as.numeric(gsub("[^0-9]", "", pday$ID))

yday$ID <- as.numeric(gsub("[^0-9]", "", yday$ID))

day <- merge(yday, pday, by = c("ID","Day"), all = TRUE)

```

# Create between-subjects and within-person means

```{r}

# Create between-subjects means (i.e., subject-specific means)

day$pdPARpos_mean <- with(day, ave(pdPARpos, ID, FUN=function(x) mean(x, na.rm=T)))

day$ydEMO_mean <- with(day, ave(ydEMO, ID, FUN=function(x) mean(x, na.rm=T)))

day$ydPA_mean <- with(day, ave(ydPA, ID, FUN=function(x) mean(x, na.rm=T)))

day$ydANGR_mean <- with(day, ave(ydANGR, ID, FUN=function(x) mean(x, na.rm=T)))

day$ydANX_mean <- with(day, ave(ydANX, ID, FUN=function(x) mean(x, na.rm=T)))

day$ydDEP_mean <- with(day, ave(ydDEP, ID, FUN=function(x) mean(x, na.rm=T)))

day$ydLSAT_mean <- with(day, ave(ydLSAT, ID, FUN=function(x) mean(x, na.rm=T)))

# center

dailyshort <- day[!duplicated(day$ID),]

describe (dailyshort$pdPARpos_mean, na.rm=TRUE) #6.443

describe (dailyshort$ydEMO_mean, na.rm=TRUE) #7.265

describe (dailyshort$ydPA_mean, na.rm=TRUE) #7.045

describe (dailyshort$ydANGR_mean, na.rm=TRUE) #2.37

describe (dailyshort$ydANX_mean, na.rm=TRUE) #2.132

describe (dailyshort$ydDEP_mean, na.rm=TRUE) #1.674

describe (dailyshort$ydLSAT_mean, na.rm=TRUE) #6.955

day$pdPARpos_between <- day$pdPARpos_mean - 6.443

day$ydEMO_between <- day$ydEMO_mean - 7.265

day$ydPA_between <- day$ydPA_mean - 7.045

day$ydANGR_between <- day$ydANGR_mean - 2.37

day$ydANX_between <- day$ydANX_mean - 2.132

day$ydDEP_between <- day$ydDEP_mean - 1.674

day$ydLSAT_between <- day$ydLSAT_mean - 6.955

# Create within-subjects means (i.e., deviation from subject-specific means)

day$pdPARpos_within <- day$pdPARpos - day$pdPARpos_mean

day$ydEMO_within <- day$ydEMO - day$ydEMO_mean

day$ydPA_within <- day$ydPA - day$ydPA_mean

day$ydANGR_within <- day$ydANGR - day$ydANGR_mean

day$ydANX_within <- day$ydANX - day$ydANX_mean

day$ydDEP_within <- day$ydDEP - day$ydDEP_mean

day$ydLSAT_within <- day$ydLSAT - day$ydLSAT_mean

```

# create prior-day mood and School-day

```{r}

day <- day %>%

arrange(ID, Day) %>%

group_by(ID) %>%

mutate(

ydPA_lag = lag(ydPA),

ydANGR_lag = lag(ydANGR),

ydANX_lag = lag(ydANX),

ydDEP_lag = lag(ydDEP),

ydLSAT_lag = lag(ydLSAT)

) %>%

ungroup()

# 1 = yes; 0 = no

day$ydSCHOOL <- ifelse(day$ydSCH0 == 2, 1, ifelse(day$ydSCH0 == 3, 0, NA))

```

# Combine daily data and baseline dataset

```{r}

pbaseline <- read.spss("ERA Baseline Survey-Parent.sav", to.data.frame = T)

ybaseline <- read.spss("ERA Baseline Survey-Youth.sav", to.data.frame = T)

pbaseline$ID <- as.numeric(gsub("[^0-9]", "", pbaseline$ID))

ybaseline$ID <- as.numeric(gsub("[^0-9]", "", ybaseline$ID))

sub_pbaseline <- select(pbaseline, ID, pbINC)

sub_ybaseline <- select(ybaseline, ID, ybAGE,ybSEX)

# recode family income

pbINC_mapping <- c(

"Less than $10,000" = 1,

"$10,000 - $19,999" = 2,

"$20,000 - $29,999" = 3,

"$30,000 - $39,999" = 4,

"$40,000 - $49,999" = 5,

"$50,000 - $59,999" = 6,

"$60,000 - $69,999" = 7,

"$70,000 - $79,999" = 8,

"$80,000 - $89,999" = 9,

"$90,000 - $99,999" = 10,

"$100,000 - $124,999" = 11,

"$125,000 or more" = 12

)

sub_pbaseline$pbINCre <- as.numeric(pbINC_mapping[as.character(sub_pbaseline$pbINC)])

# recode youth sex (female =0; male = 1)

ybSEX_mapping <- c(

"Female" = 0,

"Male" = 1

)

sub_ybaseline$ybSEXre <- as.numeric(ybSEX_mapping[as.character(sub_ybaseline$ybSEX)])

sub_ybaseline$ybSEXre <- as.factor(sub_ybaseline$ybSEXre)

sub_ybaseline$ybAGE <- as.numeric(sub_ybaseline$ybAGE)

# Parent ID = xxxxp; child ID = xxxxc; so we should remove the last character

sub_pbaseline$ID <- as.numeric(gsub("[^0-9]", "", sub_pbaseline$ID))

sub_ybaseline$ID <- as.numeric(gsub("[^0-9]", "", sub_ybaseline$ID))

# Merge baseline and daily data

day <- merge(day, sub_pbaseline, by = "ID", all.x = TRUE)

day <- merge.data.frame(day, sub_ybaseline, by='ID', all.x = TRUE)

# centering DAY of study at middle

day$dayC = day$Day - 10.5

```

# Main Effect of PBS

## Anger

```{r}

ctrl <- lmeControl(opt = 'optim')

day$ybSEXre <- as.factor(day$ybSEXre)

day$ydSCHOOL <- as.factor(day$ydSCHOOL)

## Anger

ANGR1a <- lme(fixed=ydANGR ~ dayC+ydSCHOOL+ydANGR_lag+pdPARpos_within+

pdPARpos_between+ybAGE+ybSEXre+pbINCre,

data=day,

random=~ 1+pdPARpos_within| ID,

na.action=na.omit, correlation = corAR1(),control=ctrl)

VarCorr(ANGR1a)

standardize_parameters(ANGR1a)

summary(ANGR1a)

summary_model <- summary(ANGR1a)

p_values <- summary_model$tTable[, "p-value"]

p_adjusted_fdr <- p.adjust(p_values, method = "fdr")

```

## Anxiety

```{r}

ANX1a <- lme(fixed=ydANX ~ dayC+ydSCHOOL+ydANX_lag+pdPARpos_within+

pdPARpos_between+ybAGE+ybSEXre+pbINCre,

data=day,

random=~ 1+ pdPARpos_within| ID,

na.action=na.omit, correlation = corAR1(),control=ctrl)

summary(ANX1a)

VarCorr(ANX1a)

standardize_parameters(ANX1a)

summary_model <- summary(ANX1a)

p_values <- summary_model$tTable[, "p-value"]

p_adjusted_fdr <- p.adjust(p_values, method = "fdr")

```

## Depression

```{r}

DEP1a <- lme(fixed=ydDEP ~ dayC+ydSCHOOL+ydDEP_lag+pdPARpos_within+

pdPARpos_between+ybAGE+ybSEXre+pbINCre ,

data=day,

random=~ 1+ pdPARpos_within| ID,

na.action=na.omit, correlation = corAR1(), control=ctrl)

summary(DEP1a)

VarCorr(DEP1a)

standardize_parameters(DEP1a)

summary_model <- summary(DEP1a)

p_values <- summary_model$tTable[, "p-value"]

p_adjusted_fdr <- p.adjust(p_values, method = "fdr")

```

## Positive Mood

```{r}

PA1a <- lme(fixed=ydPA ~ dayC+ydSCHOOL+ydPA_lag+pdPARpos_within+

pdPARpos_between+ybAGE+ybSEXre+pbINCre ,

data=day,

random=~ 1+ pdPARpos_within| ID,

na.action=na.omit, correlation = corAR1(), control=ctrl)

summary(PA1a)

VarCorr(PA1a)

standardize_parameters(PA1a)

summary_model <- summary(PA1a)

p_values <- summary_model$tTable[, "p-value"]

p_adjusted_fdr <- p.adjust(p_values, method = "fdr")

```

## Life Satisfaction

```{r}

LSAT1a <- lme(fixed=ydLSAT ~ dayC+ydSCHOOL+ydLSAT_lag+pdPARpos_within+

pdPARpos_between+ybAGE+ybSEXre+pbINCre,

data=day,

random=~ 1+pdPARpos_within| ID,

na.action=na.omit, correlation = corAR1(), control=ctrl)

summary(LSAT1a)

VarCorr(LSAT1a)

standardize_parameters(LSAT1a)

summary_model <- summary(LSAT1a)

p_values <- summary_model$tTable[, "p-value"]

p_adjusted_fdr <- p.adjust(p_values, method = "fdr")

```

# Interaction Model – Daily ER

## Anger

```{r}

ANGR1b <- lme(fixed=ydANGR ~ dayC+ydSCHOOL+ydANGR_lag+pdPARpos_within+ydEMO_within+pdPARpos_within*ydEMO_within+

pdPARpos_between+ydEMO_between+ybAGE+ybSEXre+pbINCre ,

data=day,

random=~ 1+ pdPARpos_within+ydEMO_within| ID,

na.action=na.omit, correlation = corAR1(), control=ctrl)

summary(ANGR1b)

VarCorr(ANGR1b)

standardize_parameters(ANGR1b)

simple_slopes(ANGR1b)

summary_model <- summary(ANGR1b)

p_values <- summary_model$tTable[, "p-value"]

p_adjusted_fdr <- p.adjust(p_values, method = "fdr")

```

## Anxiety

```{r}

ctrl <- lmeControl(opt = 'optim')

ANX1b <- lme(fixed=ydANX ~ dayC+ydSCHOOL+ydANX_lag+

pdPARpos_within+ydEMO_within+pdPARpos_within*ydEMO_within+

pdPARpos_between+ydEMO_between+ybAGE+ybSEXre+pbINCre ,

data=day,

random=~ 1+ pdPARpos_within+ydEMO_within| ID,

na.action=na.omit, correlation = corAR1(), control=ctrl)

summary(ANX1b)

VarCorr(ANX1b)

standardize_parameters(ANX1b)

summary_model <- summary(ANX1b)

p_values <- summary_model$tTable[, "p-value"]

p_adjusted_fdr <- p.adjust(p_values, method = "fdr")

```

## Depression

```{r}

DEP1b <- lme(fixed=ydDEP ~ dayC+ydSCHOOL+ydDEP_lag+

pdPARpos_within+ydEMO_within+pdPARpos_within*ydEMO_within+

pdPARpos_between+ydEMO_between+ybAGE+ybSEXre+pbINCre ,

data=day,

random=~ 1+ pdPARpos_within+ydEMO_within| ID,

na.action=na.omit, correlation = corAR1(), control=ctrl)

summary(DEP1b)

VarCorr(DEP1b)

standardize_parameters(DEP1b)

simple_slopes(DEP1b)

summary_model <- summary(DEP1b)

p_values <- summary_model$tTable[, "p-value"]

p_adjusted_fdr <- p.adjust(p_values, method = "fdr")

```

## Positive Mood

```{r}

PA1b <- lme(fixed=ydPA ~ dayC+ydSCHOOL+ydPA_lag+

pdPARpos_within+ydEMO_within+pdPARpos_within*ydEMO_within+

pdPARpos_between+ydEMO_between+ybAGE+ybSEXre+pbINCre ,

data=day,

random=~ 1+ pdPARpos_within+ydEMO_within| ID,

na.action=na.omit, correlation = corAR1(), control=ctrl)

summary(PA1b)

VarCorr(PA1b)

standardize_parameters(PA1b)

simple_slopes(PA1b)

summary_model <- summary(PA1b)

p_values <- summary_model$tTable[, "p-value"]

p_adjusted_fdr <- p.adjust(p_values, method = "fdr")

```

## Life Satisfaction

```{r}

LSAT1b <- lme(fixed=ydLSAT ~dayC+ydSCHOOL+ydLSAT_lag+ pdPARpos_within+ydEMO_within+pdPARpos_within*ydEMO_within+

pdPARpos_between+ydEMO_between+ybAGE+ybSEXre+pbINCre ,

data=day,

random=~ 1+ pdPARpos_within+ydEMO_within| ID,

na.action=na.omit, correlation = corAR1(), control=ctrl)

summary(LSAT1b)

VarCorr(LSAT1b)

standardize_parameters(LSAT1b)

simple_slopes(LSAT1b)

summary_model <- summary(LSAT1b)

p_values <- summary_model$tTable[, "p-value"]

p_adjusted_fdr <- p.adjust(p_values, method = "fdr")

```

# Interaction Model - JohnsonNeyman

```{r}

library(lmerTest)

library(interactions)

library(ggplot2)

day$ybSEXre <- as.factor(day$ybSEXre)

day$ydSCHOOL <- as.factor(day$ydSCHOOL)

day$Emotion_Regulation <- day$ydEMO_within

## Anger

ANGR1b <- lmer(ydANGR ~ dayC+ydSCHOOL+ydANGR_lag +pdPARpos_within+Emotion_Regulation+pdPARpos_within*Emotion_Regulation+

pdPARpos_between+ydEMO_between+ybAGE+ybSEXre+pbINCre + (1+Emotion_Regulation+pdPARpos_within| ID),

data=day,

na.action=na.omit,REML=TRUE)

summary(ANGR1b)

plot1 <- johnson_neyman(ANGR1b, pred = pdPARpos_within, modx = Emotion_Regulation,title = "")

plot1 <- plot1$plot + labs(

x = "Daily Positive Behavior Support (PBS)",

y = "Relations Between PBS and Angry Mood"

)

plot1

plot2 <- interact_plot(ANGR1b, pred = pdPARpos_within, modx = Emotion_Regulation,

modx.labels = c("Low ER (b = -0.07**)",

"Mean ER (b = -0.03)",

"High ER (b = 0.01)")) +

labs(x = "Daily Positive Behavior Support",

y = "Angry Mood",

color = "Emotion Regulation\n(within-person)") +

theme_classic()

plot2

## Depression

DEP1b <- lmer(ydDEP ~ dayC+ydSCHOOL+ydDEP_lag+

pdPARpos_within+Emotion_Regulation+pdPARpos_within*Emotion_Regulation+

pdPARpos_between+ydEMO_between+ybAGE+ybSEXre+pbINCre + (1+ pdPARpos_within+Emotion_Regulation| ID),

data=day,

na.action=na.omit,REML=TRUE)

summary(DEP1b)

plot3 <- johnson_neyman(DEP1b, pred = pdPARpos_within, modx = Emotion_Regulation,title = " ")

plot3 <- plot3$plot + labs(

x = "Daily Positive Behavior Support (PBS)",

y = "Relations Between PBS and Depressed Mood"

)

plot3

plot4 <- interact_plot(DEP1b, pred = pdPARpos_within, modx = Emotion_Regulation,

modx.labels = c("Low ER (b = -0.06**)",

"Mean ER (b = -0.02)",

"High ER (b = 0.01)")) +

labs(x = "Daily Positive Behavior Support",

y = "Depressed Mood",

color = "Emotion Regulation\n(within-person)") +

theme_classic()

plot4

## Positive Mood

PA1b <- lmer(ydPA ~ ydSCHOOL+ydPA_lag+

pdPARpos_within+Emotion_Regulation+pdPARpos_within*Emotion_Regulation+dayC+

pdPARpos_between+ydEMO_between+ybAGE+ybSEXre+pbINCre + (1+pdPARpos_within+Emotion_Regulation| ID),

data=day,

na.action=na.omit,REML=TRUE)

summary(PA1b)

plot5 <- johnson_neyman(PA1b, pred = pdPARpos_within, modx = Emotion_Regulation,title = " ")

plot5 <- plot5$plot + labs(

x = "Daily Positive Behavior Support (PBS)",

y = "Relations Between PBS and Positive Mood"

)

plot5

plot6 <- interact_plot(PA1b, pred = pdPARpos_within, modx = Emotion_Regulation,

modx.labels = c("Low ER (b = 0.08**)",

"Mean ER (b = 0.03)",

"High ER (b = -0.02)")) +

labs(x = "Daily Positive Behavior Support",

y = "Positive Mood",

color = "Emotion Regulation\n(within-person)") +

theme_classic()

plot6

## Life Satisfaction

LSAT1b <- lmer(ydLSAT ~ dayC+ydSCHOOL+ydLSAT_lag+

pdPARpos_within+Emotion_Regulation+pdPARpos_within*Emotion_Regulation+

pdPARpos_between+ydEMO_between+ybAGE+ybSEXre+pbINCre + (1+ pdPARpos_within+Emotion_Regulation| ID),

data=day,

na.action=na.omit,REML=TRUE)

summary(LSAT1b)

plot7 <- johnson_neyman(LSAT1b, pred = pdPARpos_within, modx = Emotion_Regulation,title = " ")

plot7 <- plot7$plot + labs(

x = "Daily Positive Behavior Support (PBS)",

y = "Relations Between PBS and Life Satisfaction"

)

plot7

plot8 <- interact_plot(LSAT1b, pred = pdPARpos_within, modx = Emotion_Regulation,

modx.labels = c("Low ER (b = 0.08**)",

"Mean ER (b = 0.02)",

"High ER (b = -0.04)")) +

labs(x = "Daily Positive Behavior Support",

y = "Life Satisfaction",

color = "Emotion Regulation\n(within-person)") +

theme_classic()

plot8

```

# Interaction Model - usual Emo

## Anger

```{r}

ANGR1c <- lme(fixed=ydANGR ~dayC+ydSCHOOL+ydANGR_lag+ pdPARpos_within+ydEMO_within+pdPARpos_within*ydEMO_between+

pdPARpos_between+ydEMO_between+ybAGE+ybSEXre+pbINCre ,

data=day,

random=~ 1+ pdPARpos_within+ydEMO_within| ID,

na.action=na.omit, correlation = corAR1(), control=ctrl)

summary(ANGR1c)

VarCorr(ANGR1c)

standardize_parameters(ANGR1c)

summary_model <- summary(ANGR1c)

p_values <- summary_model$tTable[, "p-value"]

```

## Anxiety

```{r}

ANX1c <- lme(fixed=ydANX ~dayC+ydSCHOOL+ydANX_lag+ pdPARpos_within+ydEMO_within+pdPARpos_within*ydEMO_between+

pdPARpos_between+ydEMO_between+ybAGE+ybSEXre+pbINCre ,

data=day,

random=~ 1+ pdPARpos_within+ydEMO_within| ID,

na.action=na.omit, correlation = corAR1(), control=ctrl)

summary(ANX1c)

VarCorr(ANX1c)

standardize_parameters(ANX1c)

summary_model <- summary(ANX1c)

p_values <- summary_model$tTable[, "p-value"]

p_adjusted_fdr <- p.adjust(p_values, method = "fdr")

```

## Depression

```{r}

DEP1c <- lme(fixed=ydDEP ~dayC+ydSCHOOL+ydDEP_lag+ pdPARpos_within+ydEMO_within+pdPARpos_within*ydEMO_between+

pdPARpos_between+ydEMO_between+ybAGE+ybSEXre+pbINCre ,

data=day,

random=~ 1+ pdPARpos_within+ydEMO_within| ID,

na.action=na.omit, correlation = corAR1(), control=ctrl)

summary(DEP1c)

VarCorr(DEP1c)

standardize_parameters(DEP1c)

summary_model <- summary(DEP1c)

p_values <- summary_model$tTable[, "p-value"]

p_adjusted_fdr <- p.adjust(p_values, method = "fdr")

```

## Positive Mood

```{r}

PA1c <- lme(fixed=ydPA ~ dayC+ydSCHOOL+ydPA_lag+ pdPARpos_within+ydEMO_within+pdPARpos_within*ydEMO_between+

pdPARpos_between+ydEMO_between+ybAGE+ybSEXre+pbINCre ,

data=day,

random=~ 1+ pdPARpos_within+ydEMO_within| ID,

na.action=na.omit, correlation = corAR1(), control=ctrl)

summary(PA1c)

VarCorr(PA1c)

standardize_parameters(PA1c)

summary_model <- summary(PA1c)

p_values <- summary_model$tTable[, "p-value"]

p_adjusted_fdr <- p.adjust(p_values, method = "fdr")

```

## Life Satisfaction

```{r}

LSAT1c <- lme(fixed=ydLSAT ~dayC+ydSCHOOL+ydLSAT_lag+ pdPARpos_within+ydEMO_within+pdPARpos_within*ydEMO_between+

pdPARpos_between+ydEMO_between+ybAGE+ybSEXre+pbINCre ,

data=day,

random=~ 1+ pdPARpos_within+ydEMO_within| ID,

na.action=na.omit, correlation = corAR1(), control=ctrl)

summary(LSAT1c)

VarCorr(LSAT1c)

standardize_parameters(LSAT1c)

summary_model <- summary(LSAT1c)

p_values <- summary_model$tTable[, "p-value"]

p_adjusted_fdr <- p.adjust(p_values, method = "fdr")

```
